# P09 Epidemiology and antifungal susceptibility patterns of candidaemia in a tertiary care hospital in Northern India: implications for empirical therapy

**DOI:** 10.1093/jacamr/dlag102.015

**Published:** 2026-06-26

**Authors:** Amber Prasad, Minakshi Singh, Priyal Anand, Y P Mathuria, Smita Sinha, Mukesh Bairwa, Uttam Kumar Nath

**Affiliations:** Department of Microbiology, All India Institute of Medical Sciences, Rishikesh, India; Department of Microbiology, All India Institute of Medical Sciences, Rishikesh, India; Department of Microbiology, All India Institute of Medical Sciences, Rishikesh, India; Department of Microbiology, All India Institute of Medical Sciences, Rishikesh, India; Department of Community Medicine, All India Institute of Medical Sciences, Rishikesh, India; Department of Internal Medicine, All India Institute of Medical Sciences, Rishikesh, India; Department of Medical Oncology and Haematology, All India Institute of Medical Sciences, Rishikesh, India

## Abstract

**Background:**

Candidaemia remains a leading cause of healthcare-associated bloodstream infections carrying significant morbidity and mortality. Epidemiology and antifungal resistance patterns vary markedly by geography, yet data from Northern India remain limited, hampering rational empirical therapy selection.

**Objectives:**

To determine the species distribution, clinical risk factors, antifungal susceptibility patterns, and mortality outcomes of candidaemia at a tertiary care centre in Northern India.

**Methods:**

This prospective observational study enrolled 237 blood culture-confirmed candidaemia cases from October 2022 to March 2024. Species identification was performed using conventional methods and the VITEK^®^ 2 Compact system (bioMérieux) with YST identification cards running software version 9.04. Antifungal susceptibility testing was carried out using AST-YS08 cards, with results interpreted according to CLSI breakpoints (M27-Ed4 and M60-Ed2) via the VITEK^®^ 2 Advanced Expert System. Clinical data including demographics, comorbidities, risk factors, and outcomes were extracted from prospective medical records.

**Results:**

Non-albicans *Candida* species predominated, accounting for 82.7% (196/237) of isolates. *Candida tropicalis* was the most frequently isolated species (28.3%, 67/237), followed by *C. parapsilosis* (17.7%, 42/237), *C. albicans* (17.3%, 41/237), and *C. auris* (8.0%, 19/237). Males constituted 59.1% of cases (male-to-female ratio 1.44:1), and adults aged 18–59 years formed the largest group (51.1%). Major identified risk factors included prolonged broad-spectrum antibiotic use (95.4%), central venous catheter presence (89.5%), ICU admission (60.0%), and mechanical ventilation (58.2%). Overall fluconazole resistance was 15.3% (29/189 tested). Species-specific analysis revealed *C. auris* carried the highest fluconazole resistance (63.2%), followed by *C. parapsilosis* (24.4%), whilst *C. tropicalis* remained fully susceptible. Echinocandin resistance was notably low at 4.7% (8/171 tested). Overall mortality was 37.6% (89/237). *C. tropicalis* infections demonstrated the highest species-specific mortality (52.2%) despite complete fluconazole susceptibility, followed by *C. auris* (42.1%) and *C. albicans* (41.5%). Mortality was significantly associated with ICU admission (OR 4.86, *P*<0.001), mechanical ventilation (OR 4.23, *P*<0.001), neutropenia (OR 5.71, *P*<0.001), and age greater than 60 years (OR 2.58, *P*=0.002). Patients with concurrent ICU admission and mechanical ventilation had the highest mortality (68.6%), whilst those with fewer than three risk factors had significantly lower mortality (24.3%, *P*<0.001).

**Conclusions:**

The predominance of non-albicans species, particularly *C. tropicalis*, with emerging fluconazole resistance in *C. parapsilosis* and *C. auris* challenges conventional empirical azole-based strategies. The high mortality associated with *C. tropicalis* despite in vitro azole susceptibility points to virulence mechanisms beyond drug resistance. Echinocandins remain highly effective and should be strongly considered for empirical therapy in this setting. These findings underscore the urgent need for local antifungal surveillance programmes and stewardship initiatives and highlight opportunities for developing novel diagnostic and therapeutic strategies to improve outcomes in candidaemia.

